# Interesting Case of Leprosy in Bellevue Hospital

**Published:** 1865-08

**Authors:** Frank King

**Affiliations:** Member of the Class


### Interesting Case of Leprosy in Bellevue Hospital.

BY FRANK KING, MEMBER OF THE CLASS.

A patient, aged about twenty years, was admitted into the medical ward of Bellevue Hospital, in the fall of last year, suffering from leprosy, and shortly after was presented in the amphitheatre, to the medical class. He gave the following history: had been under treatment in several hospitals previous to being admitted into this; had recovered to a certain extent, but was again attacked. He was born in British Guiana, South America; his parents, as far as he was able to judge, were always perfectly healthy; they lived in a small hut, in a low, marshy district; himself had always been in reasonably good health. When about ten years of age he was vaccinated with virus taken from a negro woman, whose mother had suffered from this disease.

Appearance at the time of presentation: intellectual, but listless, and with lack of animation; pale, emaciated, and appeared to take no interest in anything around him. His fingers and hands were distorted, and nearly destitute of nails, from previous attacks of the disease. The feet, or what remained of them, were exceedingly red, and had ulcers on the stubs of the meta-tarsal bones, for when first attacked the phalanges of the toes exfoliated by degrees, and were thrown off, till now he had only left about half of the meta-tarsal bones, and what is remarkable, this discharge of necrosed bone had occurred so often that the patient could foretel a considerable time, that there would be an exfoliation even though it had healed over. In this case the patient firmly believed his disease to have originated from the vaccine virus with which he was inoculated.

Leprosy is very frequently mentioned in the Bible as often occurring among the Jews; (it is impossible for me to say whether this is of the description there mentioned or not,) but in later times we do not hear of many cases of it recorded. The cause of leprosy is a special poison, the nature of which is obscure; unwholesome food, and irregularity in taking it, exposure to extremes, intemperance, want of sufficient exercise, and fresh air, are among its exciting causes. Congenital cases of this disease, some times occur, but they are exceedingly rare. Leprosy may often recover to a certain extent, sometimes spontaneously, sometimes with medical aid, but it is most commonly a disease which lasts during the natural life of the afflicted. It is often periodical, getting well in the summer, but breaking out again in the winter. I am unable to say that the case above recorded is of this description but as he was presented in December, with the disease in progress, it is probable that it belongs to periodical cases. The treatment is mostly hygienic; proper food, taken with strict regularity, exercise in fresh air, cleanliness, and supporting medicines, constitute the staples of treatment.

